# Development of Efficient AAV2/DJ-Based Viral Vectors to Selectively Downregulate the Expression of Neuronal or Astrocytic Target Proteins in the Rat Central Nervous System

**DOI:** 10.3389/fnmol.2019.00201

**Published:** 2019-08-20

**Authors:** Charlotte Jollé, Nicole Déglon, Catherine Pythoud, Anne-Karine Bouzier-Sore, Luc Pellerin

**Affiliations:** ^1^Department of Physiology, Université de Lausanne, Lausanne, Switzerland; ^2^Laboratory of Cellular and Molecular Neurotherapies (LCMN), Department of Clinical Neurosciences, Lausanne University Hospital, University of Lausanne, Lausanne, Switzerland; ^3^LCMN, Neurosciences Research Center, Lausanne University Hospital, University of Lausanne, Lausanne, Switzerland; ^4^Centre de Résonance Magnétique des Systèmes Biologiques UMR 5536, CNRS-Université de Bordeaux, Bordeaux, France

**Keywords:** AAV2/DJ, shRNA, neurons, astrocytes, MCT2, MCT4, miR30E, barrel cortex

## Abstract

Viral vectors have become very popular to overexpress or downregulate proteins of interest in different cell types. They conveniently allow the precise targeting of well-defined tissue areas, which is particularly useful in complex organs like the brain. In theory, each vector should have its own cell specificity that can be obtained by using different strategies (e.g., using a cell-specific promoter). For the moment, there is few vectors that have been developed to alternatively target, using the same capsid, neurons and astrocytes in the central nervous system. There is even fewer examples of adeno-associated viral vectors able to efficiently transduce cells both *in vitro* and *in vivo*. The development of viral vectors allowing the cell-specific downregulation of a protein in cultured cells of the central nervous system as well as *in vivo* within a large brain area would be highly desirable to address several important questions in neurobiology. Here we report that the use of the AAV2/DJ viral vector associated to an hybrid CMV/chicken β-actin promoter (CBA) or to a modified form of the glial fibrillary acidic protein promoter (G1B3) allows a specific transduction of neurons or astrocytes in more than half of the barrel field within the rat somatosensory cortex. Moreover, the use of the miR30E-shRNA technology led to an efficient downregulation of two proteins of interest related to metabolism both *in vitro* and *in vivo*. Our results demonstrate that it is possible to downregulate the expression of different protein isoforms in a cell-specific manner using a common serotype. It is proposed that such an approach could be extended to other cell types and used to target several proteins of interest within the same brain area.

## Introduction

Viral vectors are used as tools to spatially and temporally regulate the expression of proteins in a wide range of cell types, including brain cells (Davidson et al., 2000; Burger et al., 2004). Adeno-associated viral vectors (AAVs) are one of the most commonly used for several reasons. AAVs are single-stranded DNA viruses with a short DNA length, about 4.7 kb (Hoggan et al., 1966). Because of their inability to replicate in the absence of a helper virus, e.g., Adenovirus or Herpesvirus, and because of their episomal form in the nucleus, working with AAVs in the laboratory is considered as safe (Dismuke et al., 2013). Moreover, this virus is not associated with any human disease (Flotte and Berns, 2005). It was shown that over 80% of the human population has been infected with the AAV2 (Calcedo et al., 2009). Because of their small size (about 20 nm), AAVs largely diffuse in the rodent brain and are widely used for *in vivo* brain studies (Aschauer et al., 2013). Unfortunately, most of the AAVs have a low efficiency *in vitro* (Ellis et al., 2013). This downside forced researchers in the past to choose different serotypes or even different viral vectors for *in vitro* and *in vivo* studies, introducing an unavoidable bias in their study.

Few years ago, the AAV2/DJ serotype was developed (Grimm et al., 2008). This serotype was created from the shuffling of capsids between eight existing serotypes (AAV2, 4, 5, 8, 9, Avian, Caprine, and Bovine). It was shown *in vitro* that the AAV2/DJ outperforms the other serotypes in terms of transduction efficiency *in vitro* (above 1,000 times depending on the cell line studied). It was also shown that the AAV2/DJ remains highly efficient *in vivo* like the other AAVs (Holehonnur et al., 2014; de Solis et al., 2017).

The ability to transduce different cell types is primarily determined by the AAV capsid proteins. Each AAV serotype has its own tropism, i.e., its own specificity for a particular cell type (Daya and Berns, 2008). Most AAVs have a prominent neuronal tropism (e.g., AAV2, AAV5) except AAV4 that targets predominantly astrocytes (Liu et al., 2005). Several years ago, it was shown that the capsid is not the only parameter that determines the cell specificity of a viral vector. Changing the promoter can modify the cell-specific expression of the transduced sequence, e.g., the use of an astrocyte-specific promoter can change the cell-specific expression from neuronal to astrocytic (Brenner et al., 1994).

Adeno-associated viral vectors are widely used to overexpress small proteins in a specific cell type. Fewer studies were conducted using AAVs to downregulate the expression of a protein in a cell-specific manner. Indeed, the AAV DNA length (4.7 kb) prevents the use of efficient knockout strategies such as the CRISPR-Cas9 system. The transgene size is usually too large, over 3 kb (Jiang and Doudna, 2017). The other commonly used strategy for protein expression downregulation is RNA interference, a post-transcriptional gene regulation mechanism that uses short hairpin RNAs (shRNAs) complementary to the targeted mRNA that will bind to it and favor its degradation (Rao et al., 2009). The incorporation of shRNAs into endogenous microRNA contexts is offering the possibility to use cell-type specific polymerase II promoter. Furthermore, Fellmann et al. (2013) showed that embedding the shRNA sequence in a miR30E backbone (miR30E-shRNA) allows the cell to recognize the sequence as its own and process it in a controlled manner, increasing the yield of downregulation.

The use of a highly efficient downregulation tool targeting different cell types could permit to conduct studies targeting different isoforms of the same protein family expressed by different cell types within the same tissue. The association of the miR30E-shRNA with the AAV2/DJ represents an interesting strategy to target different isoforms in different cell types in the same tissue. Our goal was to combine those two elements to downregulate, *in vitro* and *in vivo*, the expression of two monocarboxylate transporter isoforms (MCTs). These proteins are key players for the astrocyte-neuron lactate shuttle (ANLS), a mechanism purported to play a central role in neuroenergetics (Pellerin and Magistretti, 1994). MCTs form a small group of proton-linked carriers of energy substrates, which includes lactate, pyruvate and ketone bodies. In the central nervous system, MCT2 is predominantly expressed by neurons whereas MCT4 is found solely on astrocytes (Halestrap and Price, 1999).

To achieve our goal, we developed AAV2/DJ viral vectors with two different promoters specific for neurons or astrocytes, and injected them in the barrel field of the rat primary somatosensory cortex (S1BF). We verified that, as expected, the chicken β-actin promoter (CBA, 0.8 kbp; Wang et al., 2003; Gray et al., 2011) led to a neuronal expression of the reporter protein and the modified form of the glial fibrillary acidic protein promoter G1B3 (Merienne et al., 2017) led to an astrocytic expression. Then, derived vectors harboring a specific shRNA sequence against one of the two MCT isoforms were tested *in vitro* and *in vivo* for their capacity to selectively downregulate the expression of MCT2 and MCT4.

## Materials and Methods

Reference of materials and resources can be found in Supplementary Table 1.

### Animals

Adult Wistar rats (over 7 weeks old, males only for *in vivo* experiments and pregnant females for *in vitro* experiments, Janvier Laboratories, RRID:RGD_13508588) were used under the protocol approved by the Swiss “Service de la Consommation et des Affaires Vétérinaires (SCAV, authorization n°3101.1) in accordance with Swiss animal welfare laws. They were housed by two and maintained on a 12 h light/dark cycle. Food and water were provided *ad libitum* throughout the experiment. Littermates of the same sex were randomly assigned to experimental groups.

### Plasmids Cloning

Plasmids from Geneart (Supplementary Table 1, Recombinant DNA, pMK) were cloned into a pENTR (pENTR-mCherry-miR30E-shHTT6) kindly provided by Pr. Nicole Déglon (Supplementary Table 1, Recombinant DNA, pENTR). This pENTR already contained the reporter gene (mCherry). Then, Gateway LR Clonase reaction was performed to insert the pENTR fragment mCherry-miR30E-miR30E-shRNA into destination vectors pAAV2ss-CBA-RFA-WPRE-bGH or pAAV2-G1B3-RFA-WPRE-bGH. Final products (Supplementary Table 1, Recombinant DNA, pAAV2ss) were then used to produce AAVs. The CBA promoter is a hybrid promoter corresponding to the chicken beta actin promoter with the enhancer sequence of the cytomegalovirus (Wang et al., 2003; Gray et al., 2011). The G1B3 promoter is a modified form of the GfaABC1D promoter, derived from the Glial Fibrillary Acidic Protein (GFAP) (Merienne et al., 2017). More precisely, three copies of the “B” enhancer sequence from Gfa2(b)3 (De Leeuw et al., 2006) have been cloned in the GfaABC1D promoter (Lee et al., 2008) to generate GfaABC1D(B3) (hereafter called G1B3; Merienne et al., 2017). The combination of those modifications led to a greater expression level and a more astrocyte-specific expression. The ITR (Inverted Terminal Sequence) allows the formation of episomally stable concatemers. The WPRE (Woodchuck hepatitis virus Post-Regulational Element) sequence enhances the expression of the transgene. The bGH (bovine Growth Hormone) sequence promotes polyadenylation and termination of the transgene.

### AAV Production

Adeno-associated viral vectors were produced in HEK293T cells, transfected with pHelper, pAAV-DJ_Rep_Cap and pAAV2-transgene using the calcium phosphate precipitation method. Cells and supernatant were harvested 72 h post-transfection and centrifuged for 10 min at 300 *g* at 4°C. Supernatant and cell pellet were processed in parallel. Supernatant was incubated in 8% Polyethylene Glycol, 2.5 M NaCl for 2 h at 4°C. Pellets were pooled and incubated with lysis buffer (0.15 M NaCl, 50 mM Tris–HCl, pH 8.5) for three cycles of freeze/thaw steps (30 min in dry ice/ethanol followed by 30 min at 37°C). The PEG-precipitated supernatant was centrifuged at 4,000 *g* for 20 min at 4°C and the pellet was stored. The lysate was added to the pellet and incubated at 37°C for 1 h. The cellular lysate was treated with Benzonase (50 units/mL) in 1 M MgCl_2_ for 30 min at 37°C. Then the lysate was centrifuged at 4,000 *g* for 20 min at 4°C. AAVs were separated using iodixanol gradient centrifugation at 59,000 rpm (70Ti rotor, Beckman-Coulter) for 90 min at 20°C. Phase containing AAVs was harvested and loaded on an Amicon Ultra-15PL 100 column with 0.001% Pluronic F68 D-PBS for iodixanol cleaning and viral particles concentration. Tubes were first centrifuged at 4,000 *g* at 4°C until the whole solution has passed through the column. AAVs were resuspended in 200 μL 0.001% Pluronic F68 D-PBS. The viral genome content (vg/mL) of each AAV2/DJ was assessed by Taqman^®^ qPCR with primers recognizing Inverted Terminal Repeats of AAV2 viral genome (Supplementary Table 1, Oligonucleotides). AAV2/DJ was stored at −80°C until use.

### Stereotaxic Surgery

Surgeries were performed on 7 weeks old animals. Animals were randomly assigned to experimental groups. Animals were anesthetized with isoflurane (5% for the induction and 3% to maintain the anesthesia). AAVs or PBS were injected in one site/hemisphere (S1BF: Anteroposterior = −2,3 mm; Mediolateral = ±5 mm; Dorsoventral = −3 mm). Viral vectors were injected with 34 G steel cannula fixed on a cannula holder and linked to a 10 μL Hamilton syringe and an infusion pump. For each site, 4 μL of viral vector were injected at 0.2 μL/min. Cannulas were left in the brain for 5 min after the injection, and then slowly removed. Skin was closed using 4.0 sterile suture thread. Sterile NaCl 0.9% solution (1 mL) was delivered to the rat by intra-peritoneal injection to avoid dehydration after surgery, and healing cream was applied on the head. Sugar-taste Paracetamol was delivered to the animal in water (1 *g*/cage for rats) during 72 h. Animals were monitored until complete awakening, and every day during 3 days after the surgery. All viral vectors were injected at a final concentration of 1 × 10^8^ *g*/site.

### Brain Samples Processing

For brain fixation, animals were anesthetized by lethal i.p. injection of Pentobarbital (150 mg/kg, 1 mL/kg). Intra-cardiac perfusion of cold PBS 1× was performed during 1 min (30 mL/min), followed by perfusion of cold fresh Paraformaldehyde 4% (PFA 4%) solution diluted in 0.15 M of Na-Phosphate buffer during 10 min (30 mL/min). The brain was quickly dissected and post-fixed in PFA 4% during 12 h, followed by cryo-protection in PBS 1×-Sucrose 20% (24 h) and PBS 1×-Sucrose 30% (24 h). Brains were conserved at –80°C until being sectioned at 25 μm with a cryostat.

For RNA and protein extractions, all procedures were performed under RNAse-free conditions. Rats were slightly anesthetized using Isoflurane and quickly decapitated. After, brain was removed and placed in a cold dissection matrix to prepare 1 mm sections. Barrel cortex area was quickly isolated and punches were immediately homogenized on ice in 1 mL of Trizol Reagent and stored at −80°C until use.

### Mixed Primary Cultures of Rat Cortical Neurons and Astrocytes

The day before dissection, culture wells (6-well plates) were coated with Poly-L-Ornithine Hydrobromide 15 mg/mL. The pregnant female was sacrificed (at E17–17.5, days of gestation) by decapitation. Embryos were extracted and then cortices were isolated and minced. Minced cortices were then incubated at 37°C with a papain solution (HBSS with Penicillin-Streptomycin 1×, 1 mM L-cysteine, DNAse I 1,000 U and Papain 200 U) for 30 min. Papain activity was then quenched by 1 mL of FBS. Cortices were then finely dissociated in culture medium (High glucose DMEM, B-27 supplement 1×, FBS 10%) by gentle up and down movements in a sterile Pasteur pipette. The solution containing the dissociated cells was then centrifuged for 15 min at 1,000 *g*. Cells were resuspended in culture medium, counted and plated (200,000 cells per well). A neurons/astrocytes proportion of about 1:3 was obtained at the end of the culture time (because of astrocytes proliferation).

The MOI (Multiplicity of Infection) was calculated as the number of viral particles needed per cell (AAV number/total number of cells, Ellis et al., 2013). After 5 days of culture, the culture medium was changed and cells were infected using increasing doses of viral vectors (MOI of 500, 2,000, 4,000 and 7,000). To avoid differences in temperature and volume of culture medium, only 40 μL of diluted vector were added per well.

After 11 days of culture, cells were incubated with 600 μM of DETA-NONOate, a NO donor, for 16 h. This exposure mimics the effect of physoxia. Indeed, at 21% of O_2_, astrocytes do not express MCT4 (Marcillac et al., 2011). At day 12 *in vitro*, all cells were collected using 350 μL of RLT Buffer from the RNeasy mini kit (Qiagen) and stored at −80°C until use for mRNA extraction and in 100 μL of RIPA buffer and stored at −20°C until use for protein extraction.

### Immunohistochemistry

Primary and secondary antibodies used in this study are described in the Supplementary Table 1. Free-floating sections of 25 μm were washed three times (5 min/wash) at RT in PBS 1×, blocked 1 h in PBS 1× containing 10% Bovine Serum Albumin Fraction V (BSA) and 0.1% of Triton x-100. Sections were incubated overnight at 4°C in PBS 1× containing 5% BSA, 0.1% Triton x-100 and primary antibodies diluted at 1/500. The following day, sections were washed three times (5 min/wash) in PBS 1× and incubated 2 h in PBS 1× containing 5% BSA, 0.1% Triton x-100 and secondary antibodies (diluted at 1/1,000). Sections were finally washed 3 times in PBS 1×, incubated in Hoechst 33342 trihydrochloride trihydrate solution (10 μg/mL) during 5 min at RT, washed three times in PBS 1× and mounted on SuperFrost Ultra Plus microscope slide in Fluoromount medium.

### RNA and Protein Extraction

RNA and protein extractions were performed on ice under RNAse free conditions, with ultrapure sterile RNAse-DNAse-Protease free water. Extractions from *in vivo* experiments were performed according to the Trizol kit recommendations (mRNA and protein extraction). Extractions from *in vitro* experiments were performed according to the RNeasy mini kit recommendations. At the end, RNAs were finally resuspended in 22 μL of ultrapure sterile RNAse-DNAse-Protease free water and stored at −80°C. RNA concentration and potential chemical contamination were determined using a Nanodrop 1,000. Samples with aberrant 280/260 and 260/230 ratio were discarded.

Cells were collected in RIPA buffer. Samples were sonicated 3 times for 5 s at an intensity of 70% and centrifuged at 10,000 *g* for 5 min. Supernatants were collected and protein concentration was measured using a micro BCA assay (see below).

### Reverse Transcription and qPCR

Reverse transcription was performed according to the SuperScript Transcriptase II protocol. 50 μM Random Hexamers and 10 mM dNTP mix were added to 200 ng of samples. Samples were incubated for 5 min at 65°C. Then, First Strand Buffer 5×, 0.1 M DTT and ultrapure DNAse-RNAse free water were added. Samples were incubated for 2 min at 25°C. Finally, 200 units of SuperScript Transcriptase II were added and tubes were incubated in a Thermocycler to perform the reaction (10 min at 25°C, 50 min at 42°C and 15 min at 70°C). At the end, cDNAs were diluted to obtain a final concentration of 1 ng/L.

Quantitative PCR was performed on 2 ng of cDNA following the protocol of the SensiFAST SyBr Hi-Rox kit. Samples were incubated at 95°C for 3 min then 40 cycles of 3 s at 95°C and 20 s at 60°C. For every experiment, the expression of the gene of interest was reported to RPS29 (Ribosomal Protein S29) expression.

### Protein Measurement and Western Blot

Protein concentration was evaluated using the Micro BCA Protein assay kit, according to the manufacturer’s recommendations. Diluted proteins were mixed with Laemmli Buffer 4×. Samples and molecular standards (PageRuler) were loaded on a SDS-PAGE 12% acrylamide gel and then transferred on a nitrocellulose membrane by semi-dry transfer (Transblot Turbo). At the end of transfer, membranes were blocked for 1 h, under agitation, at RT with Odyssey Blocking Buffer. Then, membranes were incubated with the primary antibodies in the Odyssey Blocking Buffer overnight, at 4°C, under agitation. The following day, membranes were washed in PBS-0.1% Triton x-100 and incubated with secondary antibodies diluted in Odyssey Blocking Buffer for 2 h, at RT, under agitation. After few washes in PBS-0.1% Triton x-100, membranes were revealed with the LI-COR Odyssey device. The expression of the gene of interest was reported to β-actin expression for *in vitro* experiments and to β III-tubulin expression for *in vivo* experiments.

### Image Acquisition and Quantification

Images were obtained using a Zeiss LSM 710 Quasar confocal microscope. For diffusion analysis, all acquisition parameters were kept constant between sections for each animal. All analyses were performed on raw unmodified images. Colocalization analysis was performed on ImageJ (v1.44 p), using the Plugin Cell Counter^1^. To analyze the diffusion of the vector, Tile scans of 7 × 7 were taken on the confocal microscope.

### Statistical Analysis

Statistical analyses were performed with GraphPad Prism (v. 7.04). For colocalization analyses and *in vivo* studies, a Student’s *t*-test was applied and results were considered significant when *p* < 0.05. Results are presented as mean ± SEM. For *in vitro* studies, one-way ANOVA was used followed by a Dunnett’s test that compares each condition to the non-transduced condition.

## Results

AAV2/DJ-based viral vectors have been recently introduced and need to be further characterized before being popularized as tools for biological studies, notably in the central nervous system. Indeed, it has already been tested in the brain to overexpress a protein as a model of Huntington’s disease (Jang et al., 2018) but not to downregulate protein isoforms. In order to do so, different parameters such as their cell-specific transduction and diffusion *in vivo* were measured to validate their usefulness.

### Large Diffusion in the Rat S1BF Area and Neuron-Specific Expression of the Transgene With an AAV2/DJ Viral Vector Containing a CBA Promoter

**FIGURE 1:**
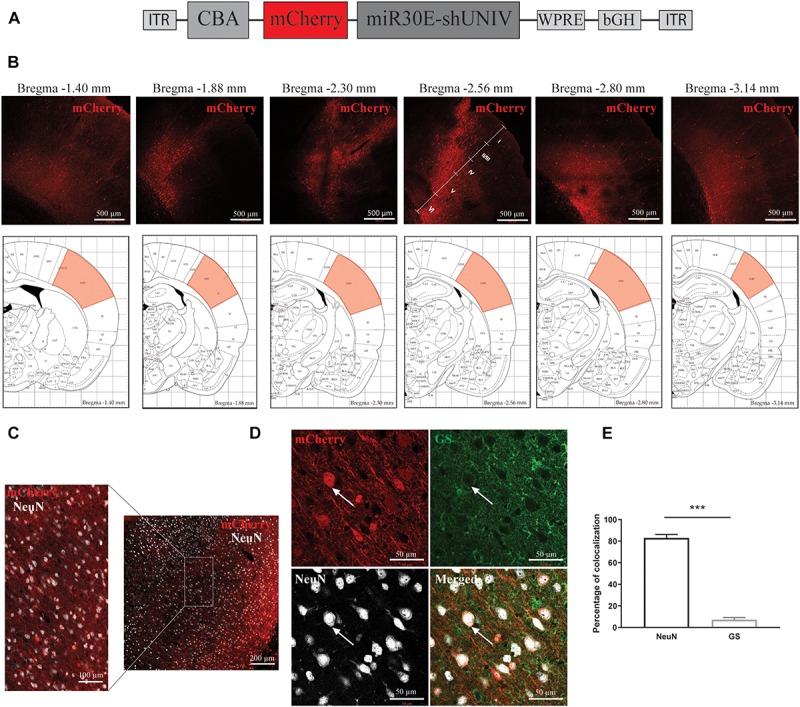
Large diffusion and neuronal specificity of the AAV2/DJ-CBA-mCherry-miR30E-shUNIV vector within the S1BF area of the rat cerebral cortex. **(A)** Schematic representation of the viral vector construction used to target neurons. The mCherry transgene was placed under the control of a CBA promoter. The shUNIV is a non-coding sequence embedded in a miR30E sequence. **(B)** Confocal mosaic pictures of the mCherry signal (reporter protein) after immunolabeling of coronal brain sections taken at 20× magnification showing the diffusion of the viral vector in the cerebral cortex along the anteroposterior axis (upper panels). The first picture was taken from a section at Bregma –1.40 mm and the last one from a section at Bregma –3.14 mm. On the fourth picture, the position of the different cortical layers is indicated. Scale bar = 500 μm. The lower panels represent schemes taken from the Paxinos rat atlas (Paxinos and Watson, 1996). Each of the six schemes corresponds to the bregma level presented in the panel above. The area colored in red corresponds to S1BF, the targeted area. **(C)** Representative confocal pictures of the transduced area in a coronal brain section submitted to a co-immunolabeling for mCherry and the neuronal marker NeuN. The right part of the panel represents a mosaic picture (5 × 5 at 20× magnification) of the transduced area in S1BF. Scale bar = 200 μm. The left part of the panel represents a portion of the transduced area at 20× magnification. Scale bar = 100 μm. **(D)** Representative confocal pictures at high magnification (40×) of S1BF 3 weeks after the injection of the viral vector and submitted to a co-immunolabeling for mCherry, NeuN and GS. The white arrow indicates a typical transduced neuron. Scale bar = 50 μm. **(E)** Quantification of the percentage of mCherry-positive/NeuN-positive cells and mCherry-positive/GS-positive cells. Data are presented as mean ± SEM. Quantification was performed on two to six images per section, six sections per animal, from two animals. Statistical analysis was performed using a Student’s *t*-test. ^∗∗∗^*p* < 0.001.

A first construct was generated with a control non-coding sequence (shUNIV) embedded in a miR30E sequence positioned after a mCherry sequence, both under the control of a CBA promoter (which was reported to promote a neuronal expression, Meunier et al., 2016; Figure 1A). The resulting AAV2/DJ-CBA-mCherry-mir30E-shUNIV viral vector was injected at a single site in the S1BF area of the rat (stereotaxic coordinates: anteroposterior = −2.3 mm; mediolateral = ±5 mm; dorsoventral = −3 mm). After 3 weeks, the diffusion of the viral vector was analyzed using immunofluorescence to amplify the mCherry signal and enhance its detection, especially in small neuronal elements such as fine processes. Transduced cell bodies were found in a large area of S1BF (±0.5 mm around the needle track along the anteroposterior axis) mainly localized in layers IV/V/VI (Figure 1B). In addition, many fibers were also transduced (Figure 1C) and covered a larger area than cell bodies (±1 mm around the needle track along the anteroposterior axis). Transduced fibers were found in all cortical layers. If all transduced cell bodies and fibers are taken into account, the viral vector has diffused over half of the entire S1BF area. The cellular specificity of the vector was determined by analyzing mCherry expression in the two major cell types transduced by the vector (Figure 1D). Co-localization between NeuN/mCherry (revealing transduced neurons) and GS/mCherry (revealing transduced astrocytes) was quantified. The vector preferentially and largely transduced neurons over astrocytes (89.44% ± 0.99 vs. 3.18% ± 0.66, respectively, Figure 1E). Moreover, with the dose used (see section “Materials and Methods”) and a single injection, the viral vector was able to transduce 34% of NeuN-positive neurons within the transduced area.

### Large Diffusion in the Rat S1BF Area and Astrocyte-Specific Expression of the Transgene With an AAV2/DJ Vector Containing a G1B3 Promoter

**FIGURE 2:**
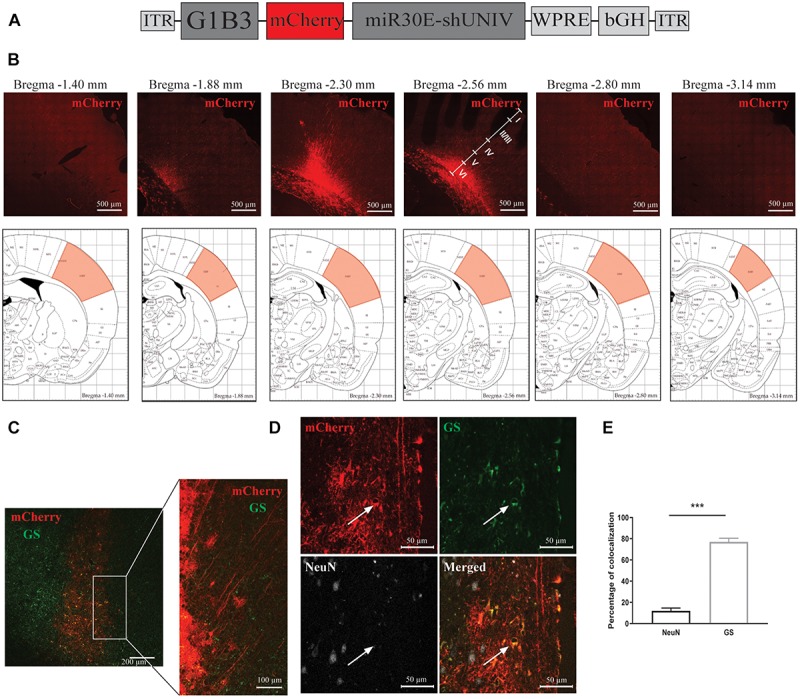
Large diffusion and astrocytic specificity of the AAV2/DJ-G1B3-mCherry-miR30E-shUNIV vector within the S1BF area of the rat cerebral cortex. **(A)** Schematic representation of the viral vector construction used to target astrocytes. The mCherry transgene was placed under the control of a G1B3 promoter. The shUNIV is a non-coding sequence embedded in a miR30E sequence. **(B)** Confocal mosaic pictures of the mCherry signal (reporter protein) after immunolabeling of coronal brain sections taken at 20× magnification showing the diffusion of the viral vector in the cerebral cortex along the anteroposterior axis (upper panels). The first picture was taken at bregma –1.40 mm and the last one at Bregma –3.14 mm. On the fourth picture, the position of the different cortical layers is indicated. Scale bar = 500 μm. The lower panels represent schemes taken from the Paxinos rat atlas (Paxinos and Watson, 1996). Each of the six schemes corresponds to the bregma level presented in the panel above. The red area corresponds to S1BF, the targeted area. **(C)** Representative confocal pictures of the transduced area in a coronal brain section submitted to a co-immunolabeling for mCherry and the astrocytic marker GS. The left panel represents a mosaic picture (5 × 5 at 20× magnification) of the transduced area in S1BF. Scale bar = 200 μm. The right picture represents a portion of the transduced area at 20× magnification. Scale bar = 100 μm. **(D)** Representative confocal pictures at high magnification (40×) of S1BF 3 weeks after the injection of the viral vector and submitted to a co-immunolabeling for mCherry, NeuN and GS. The white arrow indicates a typical transduced astrocyte. Scale bar = 50 μm. **(E)** Quantification of the percentage of mCherry-positive/NeuN-positive cells and mCherry-positive/GS-positive cells. Data are presented as mean ± SEM. Quantification was performed on two to six images per section, six sections per animal, from two animals. Statistical analysis was performed using a Student’s *t*-test. ^∗∗∗^*p* < 0.001.

A second construct was made by replacing the CBA promoter with a modified version of the astrocytic promoter GfaABC1D (Merienne et al., 2017, Figure 2A). Three copies of the B enhancer were integrated to improve transgene expression in astrocytes [GfaABC1D(B3), hereafter called G1B3] (Merienne et al., 2017). The diffusion of this newly made AAV2/DJ-G1B3-mCherry-mir30E-shUNIV viral vector was analyzed following a single injection in the S1BF area of the rat (same stereotaxic coordinates as for the AAV2/DJ-CBA-mCherry-mir30E-shUNIV above). Three weeks after the injection, detection of the mCherry signal was made by performing immunolabeling on brain sections. Transduced cell bodies were found in a restricted part of the barrel cortex (±0.2 mm around the needle track). Transduced cell bodies were found to be mainly localized in layers V/VI of the barrel cortex (Figure 2B). Many transduced processes were also visible all around cell bodies (Figure 2C). Transduced processes were found in a larger area compared to cell bodies (±1 mm around the needle track along the anteroposterior axis). All cortical layers exhibited transduced processes. Consequently, the estimated transduced area covered half of the S1BF. Again, the cellular specificity of the vector was determined by analyzing the fluorescent signal found in the two main cell types transduced by the vector (Figure 2D). Co-localization between NeuN/mCherry and GS/mCherry was quantified. The vector preferentially and largely transduced astrocytes over neurons (76.99% ± 1.72 vs. 11.99% ± 1.29, respectively, Figure 2E). With the dose used (see section “Materials and Methods”) and a single injection, approximately 70% of GS-positive cortical astrocytes were transduced by the viral vector within the transduced area.

### Efficient Downregulation of Both Neuronal MCT2 mRNA and Protein Expression *in vitro* as Well as *in vivo* Using the Same AAV2/DJ-CBA-mCherry-mir30E-shMCT2 Viral Vector

**FIGURE 3:**
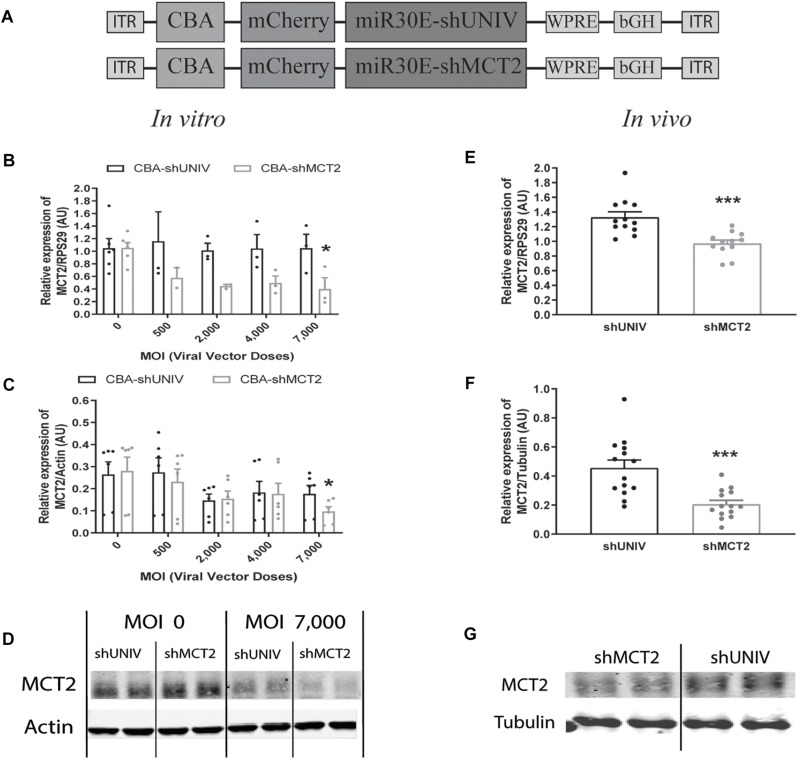
Neuronal downregulation of MCT2 expression *in vitro* and *in vivo* using an AAV2/DJ-CBA-mCherry-miR30E-shMCT2 viral vector. **(A)** Schematic representation of the control (shUNIV) and shMCT2-containing constructs used to generate the neuron-specific AAV2/DJ-based viral vectors for MCT2 invalidation experiments. The shUNIV is a non-coding sequence embedded in a miR30E sequence. The shMCT2 sequence embedded in a miR30E sequence was designed to specifically target and downregulate rat MCT2 expression. **(B–D)** Impact of AAV2/DJ-CBA-shMCT2 vector on neuronal MCT2 expression *in vitro*. Mixed primary cultures of rat cortical neurons and astrocytes were transduced with either the shUNIV-containing or the shMCT2-contining viral vector at increasing doses. MCT2 mRNA **(B)** and protein **(C)** levels have been determined by RT-qPCR and Western blot, respectively. Representative Western blots **(D)** illustrate the downregulation of MCT2 protein expression obtained at the highest viral dose with both vectors. Data are presented as mean ± SEM. *N* = 3. *n* = 6. Statistical analysis was performed using a one-way ANOVA followed by a Dunnett’s multiple comparison test for each viral condition. ^∗^*p* < 0.05 (compared to the non-transduced condition for each vector). **(E–G)** Impact of AAV2/DJ-CBA-shMCT2 on MCT2 expression *in vivo*. Rats received a bilateral single injection with either the shUNIV or the shMCT2 viral vector. MCT2 mRNA **(E)** and protein **(F)** expression have been determined by RT-qPCR and Western blot, respectively. Representative Western blots **(G)** illustrate the downregulation of MCT2 protein expression with the shMCT2 viral vector *vs.* the shUNIV viral vector. Data are presented as mean ± SEM. *N* = 7. *n* = 2. Statistical analysis was performed using a Student’s *t*-test. ^∗∗∗^*p* < 0.001. MOI, multiplicity of infection.

**FIGURE 4:**
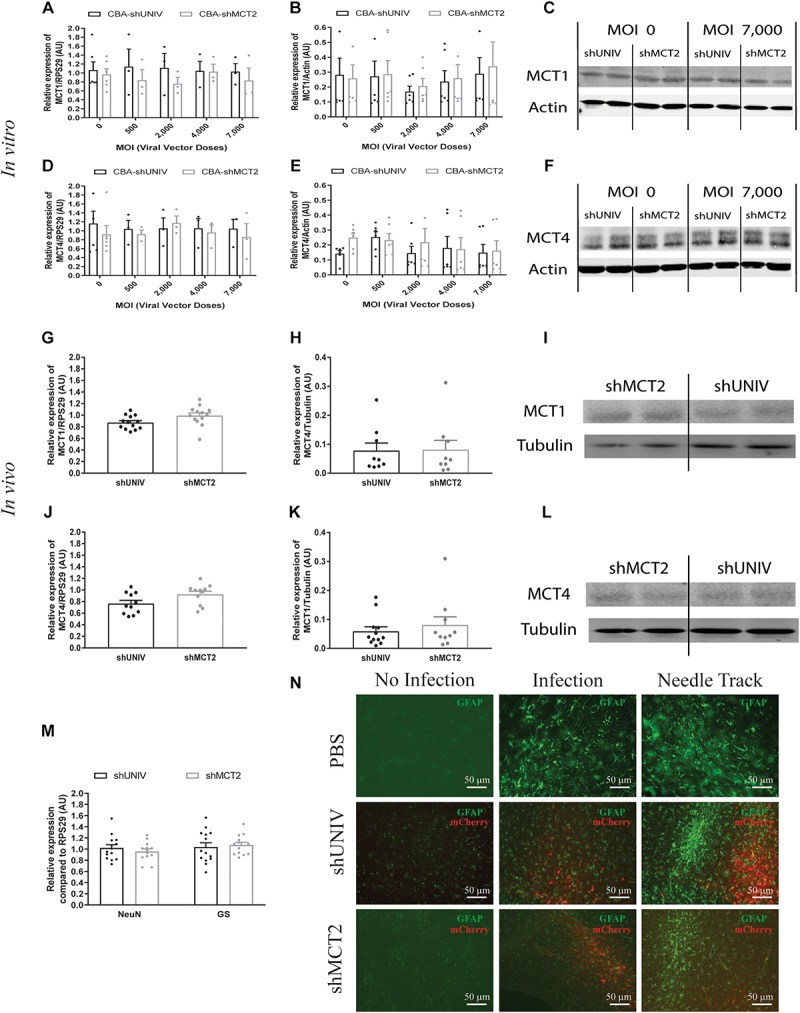
No significant effect of the AAV2/DJ-CBA-mCherry-miR30E-shMCT2 viral vector on MCT1 and MCT4 expression as well as on cell survival or astrogliosis**. (A–C)** Impact of AAV2/DJ-CBA-shMCT2 on astrocytic MCT1 expression *in vitro*. Mixed primary cultures of rat cortical neurons and astrocytes were transduced with either the shUNIV-containing or the shMCT2-containing viral vector at increasing doses. MCT1 mRNA **(A)** and protein **(B)** levels have been determined by RT-qPCR and Western blot, respectively. Representative Western blots **(C)** illustrate the lack of effect of both vectors on MCT1 protein expression. Data are presented as mean ± SEM. *N* = 3. *n* = 6. Statistical analysis was performed using a one-way ANOVA for each viral condition. **(D–F)** Impact of AAV2/DJ-CBA-shMCT2 on MCT4 expression *in vitro*. Mixed primary cultures of neurons and astrocytes were transduced with shUNIV and shMCT2 at increasing doses. Cells were also treated with DETA-NONOate at 600 μM for 16 h to induce MCT4 expression (see section “Materials and Methods”). MCT4 mRNA **(D)** and protein **(E)** levels have been determined by RT-qPCR and Western blot, respectively. Representative Western blots **(F)** illustrate the lack of effect of both vectors on MCT4 protein expression. Data are presented as mean ± SEM. *N* = 3. *n* = 6. Statistical analysis was performed using a one-way ANOVA for each viral condition. **(G–I)** Impact of AAV2/DJ-CBA-shMCT2 on MCT1 expression *in vivo*. Rats received a bilateral single injection with either the shUNIV or the shMCT2 viral vector. MCT1 mRNA **(G)** and protein **(H)** levels have been determined by RT-qPCR and Western blot, respectively. Representative Western blots **(I)** illustrate the lack of effect of both vectors on MCT1 protein expression *in vivo*. Data are presented as mean ± SEM. *N* = 7. *n* = 2. Statistical analysis was performed using a Student’s *t*-test comparing shMCT2 *vs.* shUNIV treatment. **(J–L)** Impact of AAV2/DJ-CBA-shMCT2 on MCT4 expression *in vivo*. Rats received a bilateral single injection with either the shUNIV or the shMCT2 viral vector. MCT4 mRNA **(J)** and protein **(K)** expression have been determined by RT-qPCR and Western blot, respectively. Representative Western blots **(L)** illustrate the lack of effect of both vectors on MCT4 protein expression *in vivo*. Data are represented as mean ± SEM. *N* = 7. *n* = 2. Statistical analysis was performed using a Student’s *t*-test comparing shMCT2 *vs.* shUNIV treatment. **(M)** Evaluation of neuronal and astrocytic cell death after injection of AAV2/DJ-CBA-shMCT2 in the S1BF area by measuring NeuN and GS mRNA expression. Statistical analysis was performed using a Student’s *t*-test comparing shMCT2 vs. shUNIV treatment. **(N)** Representative confocal images of immunolabeling performed against GFAP on brain coronal sections to detect astrogliosis following the injection of PBS, AAV2/DJ-CBA-shUNIV or AAV2/DJ-CBA-shMCT2 in the S1BF area. Scale bar = 50 μm. MOI, Multiplicity of infection.

A viral vector derived from the initial AAV2/DJ-CBA vector was created in order to target MCT2 in neurons. In addition to the vector used to characterize the diffusion (used here as control), one construct was made using a shRNA sequence against MCT2 (shMCT2) embedded in mir30E sequence (Figure 3A). Then, mixed primary cultures of rat cortical neurons and astrocytes were transduced with increasing doses of each viral vector named CBA-shUNIV and CBA-shMCT2 at day 5 *in vitro*. After 12 days of culture, both MCT2 mRNA and protein expression levels were determined. It was found that MCT2 mRNA expression significantly decreased with CBA-shMCT2 (reaching a decrease of 62% at the highest dose), while its expression level remained constant at all doses tested when cells were transduced with CBA-shUNIV (Figure 3B). At the protein level, both the control and the targeting vectors caused an initial decrease in expression of MCT2 at MOI 2,000 although it did not reach significance and it remained constant over doses [(*F*[4,23] = 1.252; *p* = 0.3172), Figure 3C]. However, at the highest dose, the decrease in MCT2 protein expression observed with CBA-shMCT2 was more important than with the control vector and became significant compared to the non-transduced condition (*F*[4,24] = 5.547; *p* = 0.0028). This difference between the two vectors was clearly visible on Western blots (Figure 3D). At the highest dose, the downregulation with CBA-shMCT2 reached 65% compared to the non-transduced condition. Considering the high sequence homology between MCT2 and two other MCT isoforms, MCT1 and MCT4, the specificity of the downregulation was verified by quantifying both MCT1 and MCT4 expression (Figure 4). The expression levels of both MCT1 mRNA (Figure 4A) and protein (Figure 4B) were not modified with both the shUNIV-containing and the shMCT2-containing vectors, at all doses tested. Representative Western blots illustrate the unchanged MCT1 protein levels in the different conditions (Figure 4C). The expression levels of both MCT4 mRNA (Figure 4D) and protein (Figure 4E) were unaltered with shUNIV-containing and shMCT2-containing vectors at all doses tested. Representative Western blots illustrate the unchanged MCT4 protein levels between the non-transduced condition and the highest viral dose with both vectors (Figure 4F).

The same vectors were then injected in the S1BF area of the rat brain. First, MCT2 mRNA levels were significantly decreased, by 26%, after the injection of the CBA-shMCT2 viral vector compared to the control CBA-shUNIV viral vector (Figure 3E). The downregulation at the protein level reached 54% after the injection of the CBA-shMCT2 viral vector (Figure 3F). The significant decrease of MCT2 protein expression obtained after injection of the CBA-shMCT2 viral vector was clearly visible on representative Western blots compared to the expression after treatment with the CBA-shUNIV viral vector (Figure 3G). To verify the specificity of the downregulation, expression of both MCT1 and MCT4 was quantified after the injection of either the CBA-shUNIV or the CBA-shMCT2 viral vector. No significant difference in MCT1 mRNA (Figure 4G) and protein (Figure 4H) expression was detected using each of these the two viral vectors. Representative Western blots illustrate the similar MCT1 protein levels observed with both vectors (Figure 4I). No significant difference in MCT4 mRNA (Figure 4J) and protein (Figure 4K) expression was found between the two viral vectors. Representative Western blots illustrate the lack of difference in MCT4 protein expression following the injection of the CBA-shUNIV and CBA-shMCT2 vectors (Figure 4L).

Downregulation of a protein involved in brain metabolism can cause a cellular stress and be harmful for the tissue. The presence of cell death and astrogliosis in the barrel cortex was analyzed after the injection of each viral vector in order to detect putative deleterious effects associated with the downregulation of MCT2. NeuN and GS mRNA levels were quantified to verify the absence of neuronal and astrocytic death (Figure 4M). After the injection of the CBA-shMCT2 vector, no significant difference neither in the expression of the neuronal marker (NeuN) nor in the astrocytic marker (GS) was observed compared with the injection of the control CBA-shUNIV vector. In parallel, the presence of astrogliosis was verified with GFAP staining (Figure 4N). After the injection of both viral vectors, a similar pattern of astrogliosis was observed between PBS infusion, CBA-shUNIV and CBA-shMCT2 vectors. As expected, at the site of the needle track, a strong astrogliosis occurred for both viral vectors due to mechanical damage caused by the injection itself. In non-transduced areas, no sign of astrogliosis was observed, with low expression of GFAP and astrocytes exhibiting a normal, non-hypertrophied shape. In transduced areas but away from the needle track, some reactive astrocytes (i.e., GFAP-positive and hypertrophied) were observed but with a similar occurrence for both vectors.

### Efficient Downregulation of Both Astrocytic MCT4 mRNA and Protein Expression *in vitro* as Well as *in vivo* Using the Same AAV2/DJ-G1B3-mCherry-miR30E-shMCT4 Viral Vector

**FIGURE 5:**
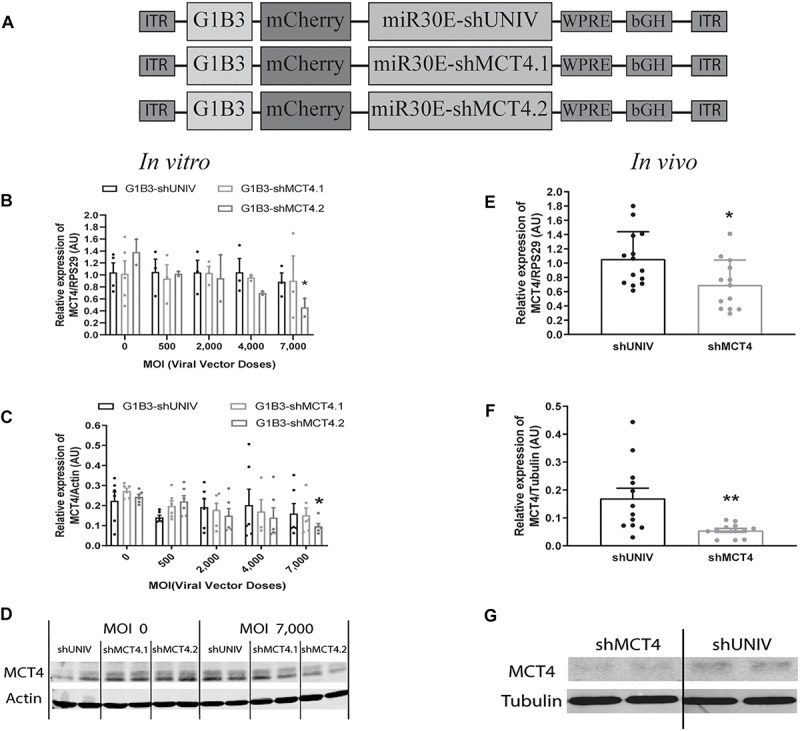
Astrocytic downregulation of MCT4 expression *in vitro* and *in vivo* using AAV2/DJ-G1B3-mCherry-miR30E-shMCT4 viral vectors. **(A)** Schematic representation of the control (shUNIV) and two shMCT4-containing constructs used to generate the astrocyte-specific AAV2/DJ-based viral vectors for MCT4 invalidation experiments. The shUNIV is a non-coding sequence embedded in a miR30E sequence. The two shMCT4 embedded in a miR30E sequence were designed to specifically target and downregulate rat MCT4 expression. **(B–D)** Impact of AAV2/DJ-G1B3-shMCT4 vectors on astrocytic MCT4 expression *in vitro*. Mixed primary cultures of rat cortical neurons and astrocytes were transduced with either the shUNIV-containing, the shMCT4.1-containing or the shMCT4.2-containing vectors at increasing doses. Cells were treated with DETA-NONOate at 600 μM for 16 h to induce MCT4 expression (see section “Materials and Methods”). MCT4 mRNA **(B)** and protein **(C)** expression have been determined by RT-qPCR and Western blot, respectively. Representative Western blots **(D)** illustrate the downregulation of MCT4 protein expression obtained with the shMCT4.2-containing vector vs. the shUNIV-containing and shMCT4.1 vectors. Data are presented as mean ± SEM. *N* = 3. *n* = 6. Statistical analysis was performed using a one-way ANOVA followed by a Dunnett’s multiple comparison test for each viral condition. ^∗^*p* < 0.05 (compared to the non-transduced condition for each vector). **(E–G)** Impact of AAV2/DJ-G1B3-shMCT4 on MCT4 expression *in vivo*. Rats received a single injection in each S1BF area with either shUNIV-containing or shMCT4.2-containing (called shMCT4) viral vector at a single dose. MCT4 mRNA **(E)** and protein **(F)** levels have been determined by RT-qPCR and Western blot, respectively. Representative Western blots **(G)** illustrate the downregulation of MCT4 protein expression with the shMCT4 viral vector *vs.* the shUNIV viral vector. Data are presented as mean ± SEM. *N* = 7. *n* = 2. Statistical analysis was performed using a Student’s *t*-test. ^∗^*p* < 0.05; ^∗∗^*p* < 0.01. MOI, Multiplicity of infection.

**FIGURE 6:**
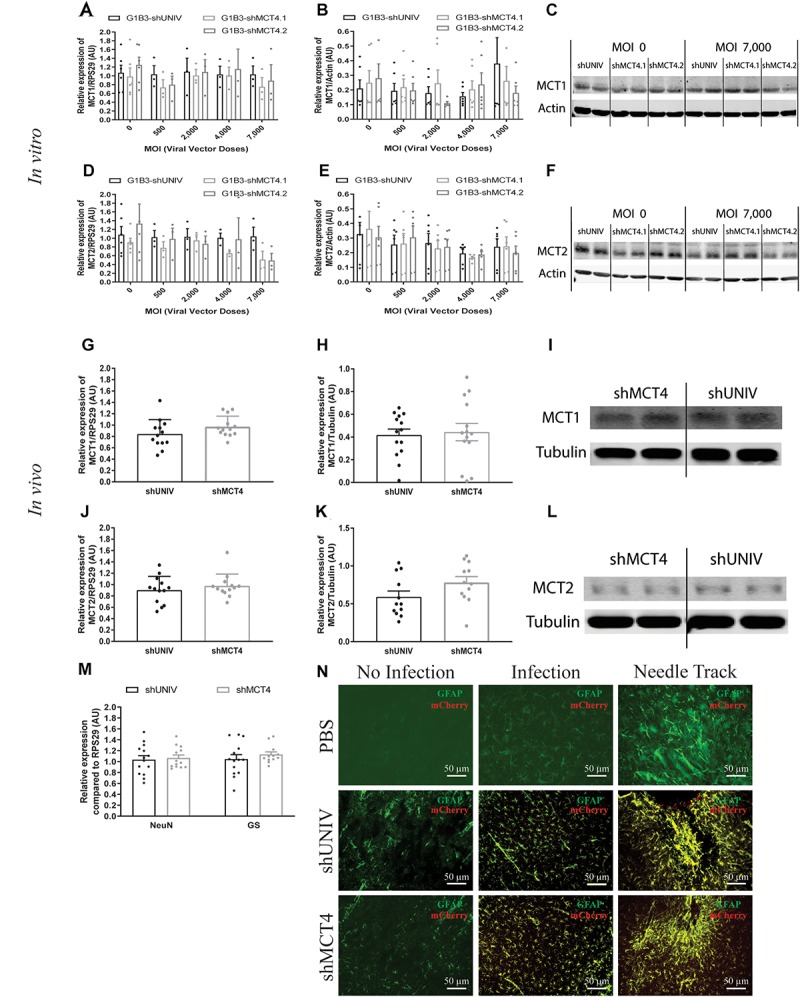
No significant effect of the AAV2/DJ-G1B3-mCherry-miR30E-shMCT4.1 and the AAV2/DJ-G1B3-mCherry-miR30E-shMCT4.2 viral vectors on MCT1 and MCT2 expression as well as on cell survival or astrogliosis. **(A–C)** Impact of AAV2/DJ-G1B3-shMCT4.1 and AAV2/DJ-G1B3-shMCT4.2 on astrocytic MCT1 expression *in vitro*. Mixed primary cultures of rat cortical neurons and astrocytes were transduced with either the shUNIV-containing, the shMCT4.1-containing or the shMCT4.2-containing viral vector at increasing doses. MCT1 mRNA **(A)** and protein **(B)** expression have been determined by RT-qPCR and Western blot, respectively. Representative Western blots **(C)** illustrate the lack of effect of the three vectors on astrocytic MCT1 protein expression. Data are presented as mean ± SEM. *N* = 3. *n* = 6. Statistical analysis was performed using a one-way ANOVA for each viral condition. **(D–F)** Impact of AAV2/DJ-G1B3-shMCT4.1 and AAV2/DJ-G1B3-shMCT4.2 on MCT1 expression *in vitro*. Mixed primary cultures of rat cortical neurons and astrocytes were transduced with either the shUNIV-containing, the shMCT4.1-containing or the shMCT4.2-containing viral vector at increasing doses. MCT2 mRNA **(D)** and protein **(E)** levels have been determined by RT-qPCR and Western blot, respectively. Representative Western blots **(F)** illustrate the lack of effect of the three vectors on neuronal MCT2 protein expression. Data are presented as mean ± SEM. *N* = 3. *n* = 6. Statistical analysis was performed using a one-way ANOVA for each viral condition. **(G–I)** Impact of AAV2/DJ-G1B3-shMCT4.2 on MCT1 expression *in vivo*. Rats received a single injection in each S1BF area with either the shUNIV-containing or the shMCT4.2-containing viral vector at a single dose. MCT1 mRNA **(G)** and protein **(H)** expression have been determined by RT-qPCR and Western blot, respectively. Representative Western blots **(I)** illustrate the lack of effect of both vectors on MCT1 protein expression *in vivo*. Data are presented as mean ± SEM. *N* = 7. *n* = 2. Statistical analysis was performed using a Student’s *t*-test comparing shMCT4.2 *vs.* shUNIV treatment. **(J–L)** Impact of AAV2/DJ-G1B3-shMCT4.2 on MCT2 expression *in vivo*. Rats received a bilateral single injection with either the shUNIV-containing or the shMCT4.2-containing viral vector. MCT2 mRNA **(J)** and protein **(K)** expression have been determined by RT-qPCR and Western blot, respectively. Representative Western blots **(L)** illustrate the lack of effect of both vectors on MCT2 protein expression *in vivo*. Data are presented as mean ± SEM. *N* = 7. *n* = 2. Statistical analysis was performed using a Student’s *t*-test comparing treatments. **(M)** Evaluation of neuronal and astrocyte cell death after injection of AAV2/DJ-G1B3-shMCT4.2 in the S1BF area by measuring NeuN and GS mRNA expression. Statistical analysis was performed using a Student’s *t*-test comparing shMCT4.2 *vs.* shUNIV treatment. **(N)** Representative confocal images of immunolabeling performed against GFAP on brain coronal sections to detect astrogliosis following the injection of PBS, AAV2/DJ-G1B3-shUNIV or AAV2/DJ-G1B3-shMCT4.2 in the S1BF area. Scale bar = 50 μm. MOI, Multiplicity of infection.

Based on the previously described vector to target astrocytes, two viral vectors were created in order to downregulate MCT4 expression in astrocytes. Two constructs were made in which a shRNA sequence against MCT4 (called either shMCT4.1 or shMCT4.2) embedded in a miR30E sequence was placed under the control of the G1B3 promoter while the original AAV2/DJ-G1B3-mCherry-miR30E-shUNIV viral vector was used as control (Figure 5A). Then, mixed primary cultures of rat cortical neurons and astrocytes were transduced at increasing doses of each viral vector. MCT4 mRNA expression levels decreased significantly and proportionally to the dose using the G1B3-shMCT4.2 vector but neither with the G1B3-shMCT4.1 vector nor with the control G1B3-shUNIV vector (Figure 5B). At the highest dose, the expression was decreased by 75%. The same profile of downregulation was found at the protein level (Figure 5C). Quantification revealed a decreased expression of the MCT4 protein proportional to the viral dose of G1B3-shMCT4.2 used, while no effect was observed with the G1B3-shMCT4.1 vector and the control G1B3-shUNIV vector. Representative Western blots illustrate the downregulation obtained at the highest dose, which reached 60% (Figure 5D). Considering the high sequence homology of MCT4 with MCT1 and MCT2, the downregulation specificity was verified by quantifying MCT1 and MCT2 expression after transduction with each viral vector (Figure 6). The levels of MCT1 mRNA (Figure 6A) and protein (Figure 6B) were not significantly modified at any dose of either G1B3-shUNIV, G1B3-shMCT4.1 or G1B3-shMCT4.2. Representative Western blots illustrate the absence of change in MCT1 protein expression at the highest dose of all vectors compared to no transduction (Figure 6C). In parallel, the levels of MCT2 mRNA (Figure 6D) and protein (Figure 6E) were unchanged for any tested dose of each viral vector. Representative Western blots illustrate the absence of modification of MCT2 protein expression with either G1B3-shUNIV, G1B3-shMCT4.1, or G1B3-shMCT4.2 at the highest dose (Figure 6F).

The control vector (G1B3-shUNIV) and the shMCT4-containing vector showing *in vitro* efficiency in downregulating MCT4 (G1B3-shMCT4.2) were then injected at a single site in the S1BF area of the rat brain (same stereotaxic coordinates as previously indicated). It was found that MCT4 mRNA expression was significantly decreased, by 34%, after the injection of the G1B3-shMCT4.2 vector compared to the shUNIV-containing control vector (Figure 5E). Downregulation of the MCT4 protein reached 67% after the injection of the G1B3-shMCT4.2 vector compared to the shUNIV-containing control (Figure 5F). Representative Western blots illustrate the significant decrease of MCT4 protein expression obtained in the S1BF area of the rat brain (Figure 5G). To verify the specificity of the downregulation obtained *in vivo*, MCT1 and MCT2 expression levels were also quantified after the injection of the G1B3-shMCT4.2 viral vector in the S1BF area. The levels of MCT1 mRNA (Figure 6G) and protein (Figure 6H) were not significantly different between the two viral vectors. Representative Western blots illustrate the absence of modification of MCT1 protein levels between both vectors (Figure 6I). Concerning MCT2, it’s mRNA (Figure 6J) and protein (Figure 6K) expression did not differ between G1B3-shMCT4.2 and G1B3-shUNIV vectors. Representative Western blots illustrate this lack of difference in MCT2 protein expression between the two vectors (Figure 6L).

Cell death and astrogliosis in the S1BF area were analyzed after the injection of either the G1B3-shMCT4.2 or the control G1B3-shUNIV viral vector. NeuN and GS mRNA levels were quantified to verify the absence of neuronal and astrocytic death, respectively (Figure 6M). No sign of neuronal death, as indicated by equivalent NeuN mRNA levels, or astrocytic death, indicated by equivalent GS mRNA levels, was observed when comparing the effect of both vectors. The presence of astrogliosis was evaluated using GFAP immunolabeling on coronal sections of the brain (Figure 6N). A similar pattern of astrogliosis was observed between PBS infusion, G1B3-shUNIV, and G1B3-shMCT4 vectors. In non-transduced areas, no astrogliosis was observed with low expression of GFAP and normal astrocytic shape. In transduced areas but away from the needle track, some reactive astrocytes were observed but their number did not differ between the two conditions. As expected at the site of the needle track, a strong astrogliosis occurred but to a similar extent for both viral vectors due to mechanical damage caused by the injection.

## Discussion

The initial goal of this study was to develop viral vectors to alternatively target two cell types by using the same capsid while modifying only the promoter of the construct. The two cell types chosen as targets were rat cortical neurons and astrocytes. Usually, viral vectors derived from either lenti- or adeno-associated viruses exhibit a preferential tropism toward either neurons or astrocytes (Wu et al., 2006; Aschauer et al., 2013). The AAV6 has already been used to selectively transduce astrocytes both *in vitro* and *in vivo* (Schober et al., 2016). However, in this study the authors used another serotype to target neurons (AAV2). Because our goal was to alternatively target a specific cell type with the same serotype, we selected the AAV2/DJ viral capsid as it was shown to efficiently transduce different cell types and cell lines (Grimm et al., 2008). Concerning the promoters, it was previously shown that the CBA promoter exhibits a neuronal specificity for expression in the brain (Burger et al., 2004; von Jonquieres et al., 2013; Meunier et al., 2016). We showed here that, in combination with an AAV2/DJ backbone, it conserved its neuronal specificity both *in vitro* and *in vivo*. The astrocytic promoter G1B3 has been shown to confer astrocyte specificity of expression in the mouse striatum (Merienne et al., 2017). Indeed, in combination with the AAV2/5, it was found to drive the expression of a transgene predominantly in astrocytes *in vivo*. The possibility to use the same viral vector to target two different cell types of the same tissue both *in vitro* and *in vivo* presents several advantages. Up to now, the use of AAV-based vectors prevented this possibility since the efficiency of infection of most AAV serotypes *in vitro* was very low (Ellis et al., 2013). It is not the case with the AAV2/DJ that permitted a significant transduction of primary cultures of both rat cortical neurons and astrocytes. It was thus possible to test *in vitro* the efficiency of our selected shRNA sequences in downregulating the expression of the target mRNAs and proteins prior to use them *in vivo*. Moreover, this approach allows to reduce the number of animals needed to assess the usefulness of such newly developed molecular tools, respecting the 3R principles (MacArthur Clarke, 2018).

In addition to their efficiency *in vitro*, the AAV2/DJ-based viral vectors were capable to efficiently transduce both neurons and astrocytes *in vivo* as demonstrated in the rat somatosensory cortex. Despite this convincing demonstration in the somatosensory cortex, it remains to be examined whether this efficiency could vary in different brain regions as previously reported for other AAV serotypes (Aschauer et al., 2013). In addition, it might be interesting to verify if this approach could be extended to other brain cell types such as oligodendrocytes or microglial cells. Although specific promoters for each of these cell types have already been identified (Goldmann et al., 2013; Georgiou et al., 2017), few AAV-based vectors have been developed to target them both *in vitro* and *in vivo*. Oligodendrocytes could be transduced *in vivo* with an AAV-based vector but it was not tested *in vitro* (von Jonquieres et al., 2013). The transduction of microglial cells *in vitro* has been reported using a modified AAV6 capsid and microglial-specific promoters but the transduction was low *in vivo* (Rosario et al., 2016). The use of the AAV2/DJ might be of interest to successfully transduce microglia and oligodendrocytes both *in vitro* and *in vivo*. Another important aspect concerns the capacity of viral vectors to diffuse over a large tissue area. Indeed, we found that AAV2/DJ-based viral vectors exhibited an important spreading capacity, sufficient to cover an area representing more than half of the entire barrel cortex with a single injection. Moreover, AAV2/DJ-based viral vectors efficiently diffused not only laterally but also throughout the cortical thickness, reaching all six cortical layers. This is also an appreciable feature since it is possible to avoid making several injections at different locations to cover the targeted area, reducing the degree of potential damage to the tissue. Interestingly, a difference in the diffusion pattern of the fluorescent signal between CBA-based and G1B3-based vectors was observed. It was recently shown that astrocytes were poorly covered by 2D imaging. Less than 10% of their true volume seemed to be covered by 2D imaging (Bindocci et al., 2017). Indeed, the soma represents only 25% of the total astrocytic volume. The diameter of a process being fine and processes being sparse, a large proportion of the processes are not captured in the focal plan. Moreover, even with the best confocal microscope, processes, and endfeet cannot be clearly seen because of resolution limitations. So, the total volume of astrocytic transduction might be underestimated although the percentage of transduced astrocytes would likely remain the same. Despite these caveats, it seems that both viral vector types based on AAV2/DJ to target alternatively neurons and astrocytes allow to express a transgene in a predominantly cell-specific manner and with a large diffusion within a brain cortical region of interest.

These new tools might be particularly useful to investigate a physiologically relevant question requiring to target the same protein or two closely related proteins in two distinct brain cell types. This is the case for two isoforms of the MCT protein family: MCT2 and MCT4. MCT2 is specifically expressed in neurons while MCT4 is exclusively expressed by astrocytes in the cortex (Pierre and Pellerin, 2005). Both transporters have been implicated in a mechanism of lactate transfer between the two cell types to ensure an adequate energy substrate supply to neurons as a function of brain activity (Pellerin and Magistretti, 2012). In order to get further insight about the role of these transporters in this mechanism, it became necessary to develop efficient and specific viral vectors to downregulate the expression of those two isoforms in rat, because working with rats rather than mice represent an advantage in certain circumstances (e.g., perform brain imaging). Using the same AAV serotype *in vitro* and *in vivo*, we show here that a significant and specific decrease in expression could be obtained for both MCT2 and MCT4. A variance of distribution is observed for the mRNA and protein quantifications. It can be explained by several reasons. First, for the *in vitro* results, the graphs summarize quantifications from several primary cultures. Even if the basal level of each mRNA/protein could differ between cultures, the degree of down regulation after transduction is the same in every culture. Then, even if the same number of cells is plated, the same dose of viral vector is given (*in vitro* as *in vitro*), leading to a certain unavoidable variability. Moreover, this downregulation was specific of the targeted isoform, since the expression of other isoforms remained unchanged. Because of technical issues related to the sampled area of tissue that cannot be limited to the transduced area and the number of infected cells that is not 100%, the degree of downregulation is underestimated *in vivo*. The use of cell sorting to isolate only transduced cells would be required to have a more precise assessment of the downregulation. Nevertheless, a significant downregulation of MCT2 and MCT4 using AAV-based viral vectors could be evidenced directly from large punches of tissue. It represents an improvement over the previous use of a classical shRNA sequence in a lentiviral vector that led to a slight decrease of the expression of MCT2 in a small fraction of cortical neurons only detectable by immunocytochemistry at the protein level (Mazuel et al., 2017). Considering that, despite the smaller decrease in MCT2 protein expression detected previously, functional effects (notably on the BOLD fMRI response during whisker stimulation) were evidenced with the lentiviral-based vector (Mazuel et al., 2017), it is likely that the new AAV-based vectors should provide more efficient tools to explore the importance of MCTs in specific brain functions.

Apart from their higher degree of transduction efficiency, and considering the high sequence homology between the different MCTs (Halestrap, 2013), the cell specificity of each AAV-based viral vector for each MCT in each cell type is another advantage. Indeed, the use of a miR30 backbone and cell-specific promoters proved to be sufficient to obtain such a specificity.

Another important issue was the possible toxicity of the AAV-based vectors. Indeed, at high doses, viral vectors can cause cell death (Colella et al., 2018; Hinderer et al., 2018). Moreover, strong and uncontrolled shRNA expression could contribute to toxicity. Although cultured astrocytes showed no sign of toxicity for the range of doses tested, we noticed that low doses of AAV2/DJ-based viral vectors caused a partial decrease of MCT2 protein expression that does not seem related to cell death of cultured neurons (since actin expression remained constant between conditions). This effect was not dose-dependent over the range of doses tested and was independent of the transgene. Although the precise reason for this effect is uncertain, primary cultures of neurons are notoriously known to be sensitive to various stressors (Fricker et al., 2018). For example, it was shown that B27-supplement deprivation of cultured cortical neurons leads to partial neuronal cell death (Alvarez-Flores et al., 2019). Cultured neurons might also be particularly sensitive to mCherry overexpression, independently of the serotype. Indeed, expression of the fluorescent reporter protein GFP has been reported to cause toxicity (Liu et al., 1999) and even neuronal death (Detrait et al., 2002). The doses that we used are considered as classical for *in vitro* transduction (Gong et al., 2004; Shevtsova et al., 2004) but cultured neurons seem to be particularly sensitive to viral vector transduction as they modify their transcriptome (Préhaud et al., 2005) or die by apoptosis (Howard et al., 2008). In our case, it seems that neurons reacted to viral transduction by modifying their proteome and decreasing MCT2. This could reflect a modification of their metabolism. Nevertheless, it was still possible in cultured neurons to unravel a significant downregulating effect of the transgene at the highest viral vector dose used. The use of a miR30E backbone for the shRNA sequence may have contributed to the absence of cell death. Indeed, it was shown that the addition of this backbone allows the cell to process the shRNA sequence through the endogenous miR30 pathways, which decreases toxicity (Boudreau et al., 2009). Consequently, it can be concluded that the observed decrease in MCT2/MCT4 expression *in vivo* is entirely due to the specific action of the shRNA sequence on the targeted mRNA.

In parallel, the reactive state of the astrocytes in the transduced area was also verified. Indeed, astrogliosis is a protective mechanism of the brain in case of CNS injuries (Sofroniew, 2015). This natural process can become deleterious when the injury or the inflammation is too high. In addition, an uncontrolled astrogliosis can alter the function of neighboring cells. An important astrogliosis just around the needle track was observed while a moderate to low astrogliosis was found in the transduced area. The astrogliosis around the needle track is essentially due to the mechanical damages caused by the needle and the liquid flow. The same degree of astrogliosis was found between PBS and the different viral vectors, highlighting the mechanical cause of this astrogliosis. In the transduced area, it could not be excluded that astrogliosis could contribute to counterbalance the downregulation of MCT4 observed with the shMCT4, thus partly masking the effective degree of MCT4 downregulation. Indeed, it was previously shown that reactive astrocytes become more glycolytic (Iglesias et al., 2017) and thus could express more MCT4, as it was shown for astrocytes *in vitro* submitted to hypoxia (Rosafio and Pellerin, 2014) or *in vivo* after ischemia (Rosafio et al., 2016).

In conclusion, unique viral tools have been successfully created that largely diffuse in the somatosensory cortex and that can specifically target neurons or astrocytes depending on the promoter used. Moreover, associated to a miR30E-shRNA strategy, an isoform-specific downregulation could be obtained with no sign of toxicity *in vivo*. In the present study, these tools have been used to target two isoforms of the MCT family, MCT2, and MCT4. Such tools will be particularly useful to study the precise role of MCTs in brain energy metabolism and brain functions *in vivo*, especially for aspects for which invalidation in rats represents an advantage over the use of transgenic mice. Until now, several studies have been conducted using pharmacological antagonists (Dimmer et al., 2000; Colen et al., 2006). To inhibit MCTs, CHC [2-Cyano-3-(4-hydroxyphenyl)-2-propenoic acid] has been widely used. However, CHC also targets other proteins such as the mitochondrial pyruvate carrier (Gray et al., 2015). To circumvent this problem, RNA interference has been used in the past (Maekawa et al., 2008; Suzuki et al., 2011), and later it was associated with a viral vector approach for an *in vivo* use. Recently, functional effects of the downregulation of MCT2 *in vivo* were detected using a lentiviral approach targeting MCT2 (Mazuel et al., 2017). However, the extent of the downregulation was limited and might preclude more complex investigations such as behavioral studies. Our new AAV-based viral vectors will not only allow to overcome these caveats but they could also be used in the future to target other proteins of interest in the central nervous system requiring a similar strategy.

## Data Availability

The datasets generated for this study are available on request to the corresponding author.

## Author Contributions

CP and ND designed and produced the viral vectors. CJ performed the *in vivo* and *in vitro* experiments. CJ and LP wrote the manuscript. CJ, ND, A-KB-S, and LP corrected the manuscript.

## Conflict of Interest Statement

The authors declare that the research was conducted in the absence of any commercial or financial relationships that could be construed as a potential conflict of interest.

## Footnotes

1 Software available on: http://imagej.nih.gov/ij/index.html.
