# Supplementary material for: Development of Efficient AAV2/DJ-Based Viral Vectors to Selectively Downregulate the Expression of Neuronal or Astrocytic Target Proteins in the Rat Central Nervous System
Source: Front Mol Neurosci. 2019 Aug 20;12:201. doi: 10.3389/fnmol.2019.00201 (PMC6710342; doi:10.3389/fnmol.2019.00201)
Supplement: Supplementary file 1 [file Table_1.docx]

Supplemental Table 1: List of reagents and resources

| REAGENT or RESOURCE | SOURCE | IDENTIFIER |
| --- | --- | --- |
| Antibodies | | |
| Goat Anti-mCherry | Sicgen | Cat# AB0040-200, RRID:AB_2333092 |
| Rabbit Anti-GS (Glutamine Synthetase) | Sigma-Aldrich | Cat# G2781, RRID:AB_259853 |
| Mouse Anti-NeuN | Millipore | Cat# MAB377, RRID:AB_2298772 |
| Rabbit Anti-GFAP | Dako | Cat# N1506, RRID:AB_10013482 |
| Goat Anti-Rabbit Cy3 | Jackson ImmunoResearch | Cat# 111-165-144, RRID:AB_2338006 |
| Donkey Anti-Mouse Cy5 | Jackson ImmunoResearch | Cat# 715-175-150, RRID:AB_2340819 |
| Donkey Anti-Goat Cy3 | Jackson ImmunoResearch | Cat# 705-166-147, RRID:AB_2340413 |
| Donkey Anti-Rabbit Alexa Fluor 488 | Jackson ImmunoResearch | Cat# 711-546-152, RRID:AB_2340619 |
| Mouse Anti-βIII-Tubulin | Sigma-Aldrich | T4026 |
| Mouse Anti-β-Actin | Sigma-Aldrich | A5441 |
| Rabbit Anti-MCT1 | Homemade | Pierre et al. (2000) Neuroscience 100:617-627 |
| Rabbit Anti-MCT2 | Homemade | Pierre et al. (2000) Neuroscience 100:617-627 |
| Rabbit Anti-MCT4 | Gift from Dr. W. Fishbein | Merezhinskaya et al (2006) Molecular Genetics and Metabolism 87: 152-161 |
| Donkey Anti-Mouse 680RD | LI-COR | 926-68072 |
| Donkey Anti-Rabbit 800CW | LI-COR | 926-32213 |
| Bacterial and Virus Strains | | |
| AAV2/DJ-CBA-mCherry-miR30E-shMCT2-WPRE-bGH | This paper | N/A |
| AAV2/DJ-CBA-mCherry-miR30E-shUNIV-WPRE-bGH | This paper | N/A |
| AAV2/DJ-G1B3-mCherry-miR30E-shUNIV-WPRE-bGH | This paper | N/A |
| AAV2/DJ-G1B3-mCherry-miR30E-shMCT4.1-WPRE-bGH | This paper | N/A |
| AAV2/DJ-G1B3-mCherry-miR30E-shMCT4.2-WPRE-bGH | This paper | N/A |
| Chemicals, Peptides, and Recombinant Proteins | | |
| DMEM High Glucose | Sigma-Aldrich | D7777 |
| Antimycotic-Antibiotic | Gibco | A5955 |
| Sodium bicarbonate | Sigma-Aldrich | S5761 |
| Fetal Bovine Serum Gamma Irradiated | Gibco | 10270-106 |
| B-27 Supplement | Gibco | 17504 |
| Gateway LR Clonase | Invitrogen | 11791-019 |
| PolyEthylene Glycol 800 | Sigma-Aldrich | N/A |
| NaCl | Sigma-Aldrich | A2942 |
| Benzonase | Sigma-Aldrich | N/A |
| Iodixanol | AxonLab | N/A |
| Pluronic F68 | Gibco | N/A |
| D-PBS | Gibco | N/A |
| Taq DNA Polymerase | Roche | N/A |
| Isoflurane | Attane Provet | N/A |
| Sterile NaCl 0.9% solution | BBraun | N/A |
| Healing Cream – Bepanthen Plus | Bayer | N/A |
| Paracetamol | Bayer | N/A |
| Pentobarbital (Esconarkon ®) | Streuli Pharma | N/A |
| Paraformaldehyde | Lucerna CHEM | 15714 |
| Sucrose | Sigma-Aldrich | S7903 |
| Poly-L-Ornithine Hydrobromide | Thomas Scientific | C979V07 |
| Penicilin-Streptomicin | Gibco | 15140-122 |
| L-Cysteina | Sigma-Aldrich | C7880 |
| DNAse I | Bioconcept | LS002138 |
| Papain | Bioconcept | LS003126 |
| DETA-NONOate | ENZO Life Science | ALX-430-014 |
| RIPA | Merck | 20-188 |
| Bovine Serum Albumin Fraction V (BSA) | Axonlab | A1391 |
| Triton | Sigma-Aldrich | x-100 |
| Hoechst 33342 Trichloride Trihydrate | Invitrogen | H3570 |
| Fluoromount medium | Sigma-Aldrich | F4680 |
| RNAse-DNAse-Protease free water | Gibco | 10977035 |
| Superscript Transcriptase II (+ First Strand Buffer and DTT) | Invitrogen | 18064-014 |
| Random Hexamers | Invitrogen | N8080127 |
| dNTP | ThermoFisher | 18427-013 |
| SensiFAST SyBr Hi-Rox Kit | Labgene | BIO92005 |
| Laemni Buffer 4x | Biorad | 161-0747 |
| PageRuler Prestained Protein | Thermofisher | 26616 |
| Odyssey Blocking Buffer | LI-COR | 927-40000 |
| Critical Commercial Assays | | |
| Trizol | Lifetechnology | 15599018 |
| RNeasy mini kit | Qiagen | 74106 |
| Micro BCA Protein Assay Kit | ThermoFisher | 23235 |
| Experimental Models: Cell Lines | | |
| Rat: primary mixed cortical neurons and astrocytes | This paper | N/A |
| Cell Line: HEK293T | This paper | N/A |
| Experimental Models: Organisms/Strains | | |
| Rat: Wistar | JANVIER Labs | RjHan:WI |
| Oligonucleotides | | |
| **shRNA sequences** |  |  |
| shUNIV: TGTATCGATCACGAGACTAGC | This paper | N/A |
| shMCT2: TAGGATTAATAGCCAACACTA | This paper | N/A |
| shMCT4.1: GCAGAAGCATTATCCAGATCT | This paper | N/A |
| shMCT4.2: GGTGAGCTATGCTAAGGATAT | This paper | N/A |
| **qPCR primers** |  |  |
| ITR-AAV-F: GGAACCCCTAGTGATGGAGTT | This paper | N/A |
| ITR-AAV-R: CGGCCTCAGTGAGCGA | This paper | N/A |
| ITR-AAV-Probe: CACTCCCTCTCTGCGCGCTCG | This paper | N/A |
| RPS29-F: GGCTTTTAGGATGGCAAGGGAC | This paper | N/A |
| RPS29-R: TGAAGGTGACAGCAGTCGGTTG | This paper | N/A |
| MCT1-F: GGAGCCAGGGTAGAGAGGAA | This paper | N/A |
| MCT1-R: TGTCATTGGAGGTCTTGGGC | This paper | N/A |
| MCT2-F: GCTCCGTATGCTAAGGAC | This paper | N/A |
| MCT2-R: CGATAGTGACGAGCCC | This paper | N/A |
| MCT4-F: GCTGTTTCATCATCACGGGC | This paper | N/A |
| MCT4-R: CATAGCCAGCAGGATGGAGG | This paper | N/A |
| NeuN-F: TCCCGAATTGCCCGAACAT | This paper | N/A |
| NeuN-R: CAACTCCACCCTTCCGACCC | This paper | N/A |
| GS-F: TCCAGATAGGACCCTGCGAA | This paper | N/A |
| GS-R: GCTAAAGTTGGTGTGGCAGC | This paper | N/A |
| Recombinant DNA | | |
| **pMK plasmids** |  |  |
| pMK-miR30E-shUNIV | This paper | N/A |
| pMK-miR30E-shMCT2 | This paper | N/A |
| pMK-miR30E-shMCT4.1 | This paper | N/A |
| pMK-miR30E-shMCT4.2 | This paper | N/A |
| **pENTR plasmids** |  |  |
| pENTR-mCherry-miR30E-shUNIV | This paper | N/A |
| pENTR-mCherry-miR30E-shMCT2 | This paper | N/A |
| pENTR-mCherry-miR30E-shMCT4.1 | This paper | N/A |
| pENTR-mCherry-miR30E-shMCT4.2 | This paper | N/A |
| **pAAV2ss plasmids** |  |  |
| pAAV2ss-ITR-G1B3-mCherry-miR30E-shUNIV-WPRE-bGH-ITR | This paper | N/A |
| pAAV2ss-ITR-G1B3-mCherry-miR30E-shMCT4.1-WPRE-bGH-ITR | This paper | N/A |
| pAAV2ss-ITR-G1B3-mCherry-miR30E-shMCT4.2-WPRE-bGH-ITR | This paper | N/A |
| pAAV2ss-ITR-CBA-mCherry-miR30E-shUNIV-WPRE-bGH-ITR | This paper | N/A |
| pAAV2ss-ITR-CBA-mCherry-miR30E-shMCT2-WPRE-bGH-ITR | This paper | N/A |
| Other | | |
| 70Ti Rotor | Beckman-Coulter | N/A |
| Amicon Ultra-15PL 100 column | Millipore | N/A |
| 34G Steel Cannulas | Unimed | N/A |
| Stereotaxic frame | Stoeling | N/A |
| 10uL Hamilton seringe | Hamilton Biomedical | N/A |
| Infusion pump | CMA | N/A |
| Suture Threas Novosyn 4.0 DS12 | BBraun | N/A |
| Transblot Turbo | BioRad | N/A |
